# Supplementary material for: Differentiation of Salivary Gland and Salivary Gland Tumor Tissue via Raman Imaging Combined with Multivariate Data Analysis
Source: Diagnostics (Basel). 2023 Dec 30;14(1):92. doi: 10.3390/diagnostics14010092 (PMC10795677; doi:10.3390/diagnostics14010092)
Supplement: Supplementary file 1 [file diagnostics-14-00092-s001.zip › diagnostics-2713017-supplementary.pdf]

Supplementary information

Overall Raman mean spectra of normal salivary gland (green), Warthin tumor (blue) and pleomorphic adenoma (red) are illustrated within 900 – 1700 cm^{-1} in Figure S1. The superposition of all three spectra shows the great resemblance and the high number of identical Raman bands. Only small differences between the tissue types can be recognized.

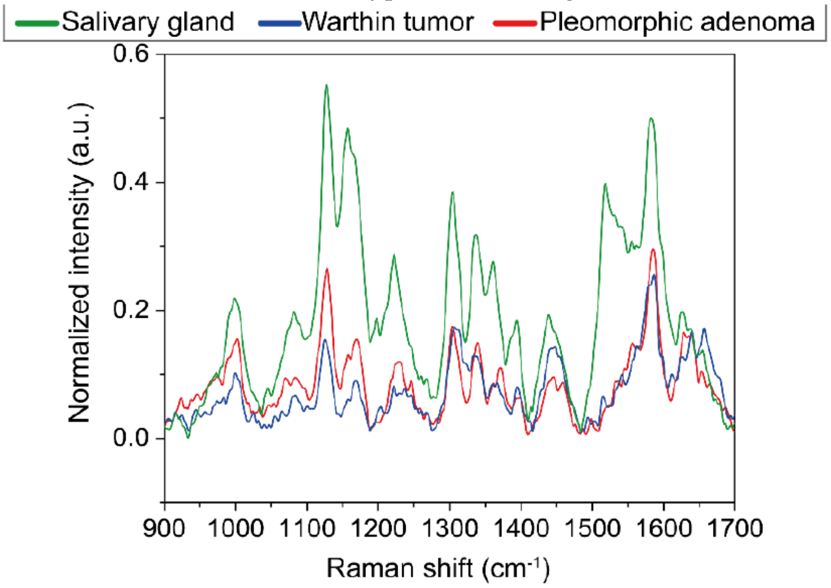

Figure S1. Superimposed Raman mean spectra of normal salivary gland (green), Warthin tumor (blue) and pleomorphic adenoma (red) within 900 – 1700 cm^{-1}. The spectral pattern of all three tissue types is very similar. Only minor changes in band relations or manifestations are noticeable.

To demonstrate the clear differentiation between parotid tissue clusters by the PCA model, various perspectives on the 3D scores plot are shown in **Figure S2**. The different angles of view reveal that the tissue groups are distinctly separated from one another with only minor overlaps at the center, independently of the plot orientation. Additionally, all plot perspectives display the necessity of PC1, PC2 and PC5 to achieve a good cluster segregation.

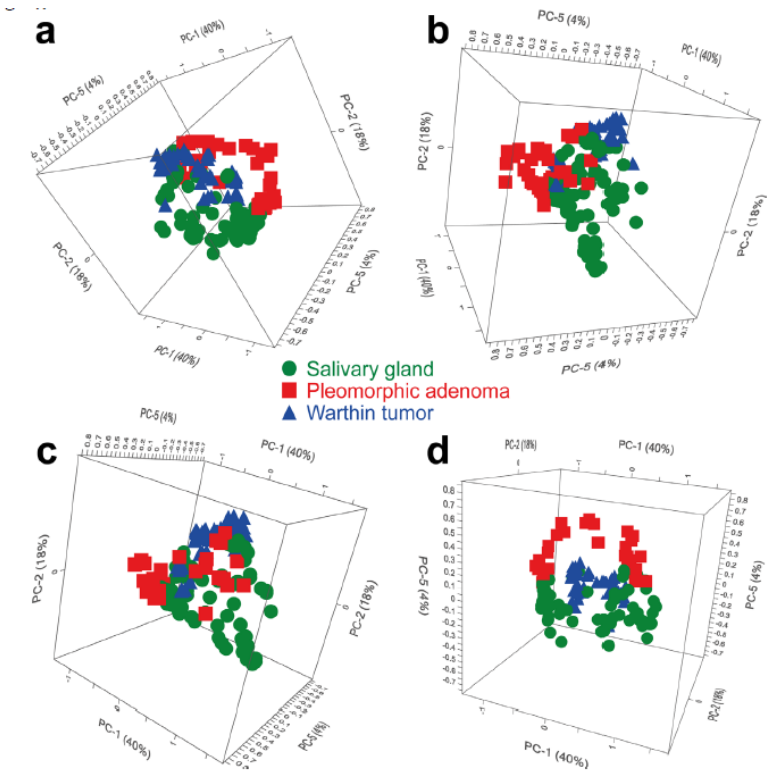

Figure S2. Different perspectives of the 3D PCA scores plot for distinguishing salivary gland, Warthin tumor and pleomorphic adenoma. A distinct separation of salivary gland (green dots), Warthin tumor (blue triangles) and pleomorphic adenoma (red boxes) is achieved by PC1 (40 %), PC2 (18 %) and PC5 (4 %) with minor overlaps between the groups. The varying angles of view on the 3D scores plot confirm the cluster segregation to be independent of the plot orientation.

In **Figure S3**, the complete PCA evaluation, represented with all used PCs (PC1 – PC5), is illustrated. The respective 2D score plots (a, c, e, g) and corresponding loadings (b, d, f, h) of PC1 – PC5 are displayed. The requirement of five PCs corroborates the complexity of the spectral data and the large information content obtained by Raman imaging.

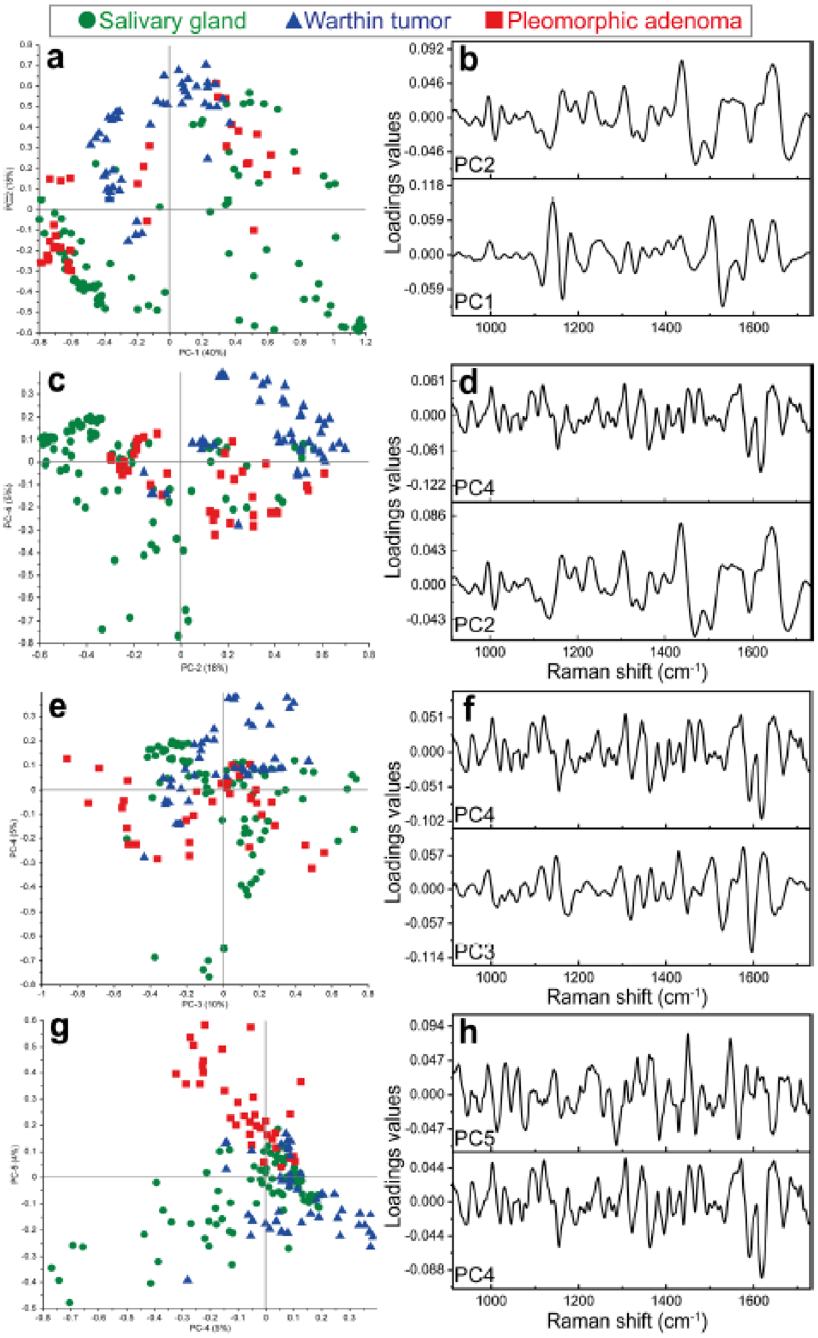

Figure S3. 2D score plots and corresponding loading plots of the PCA model (**a**-**h**). Five PCs (PC1-PC5) are required to enable a complete differentiation of normal salivary gland (green circles), Warthin tumor (blue triangles) and pleomorphic adenoma (red squares). PC1 describes 40 % of the total model variance, whereas PC2, PC3, PC4 and PC5 account for 18 %, 10 %, 5 % and 4 %, respectively. Two consecutive PCs are always plotted against one another in the 2D score plots, which are PC2 vs. PC1 (**a**) with related loading plot (**b**), PC3 vs. PC2 (**c**) with related loading plot (**d**), PC4 vs. PC3 (**e**) with related loading plot (**f**) and PC5 vs. PC4 (**g**) with related loading plot (**h**).

In **Figure S4**, score plots for the calibration and the validation model are shown, which were used for PCA evaluation. The score plots were used to estimate the model's performance. Additionally, the number of PCs were chosen by the total explained variance 75% (**Figure S4e**) and what kind of information is given by the loadings (**Figure S3**).

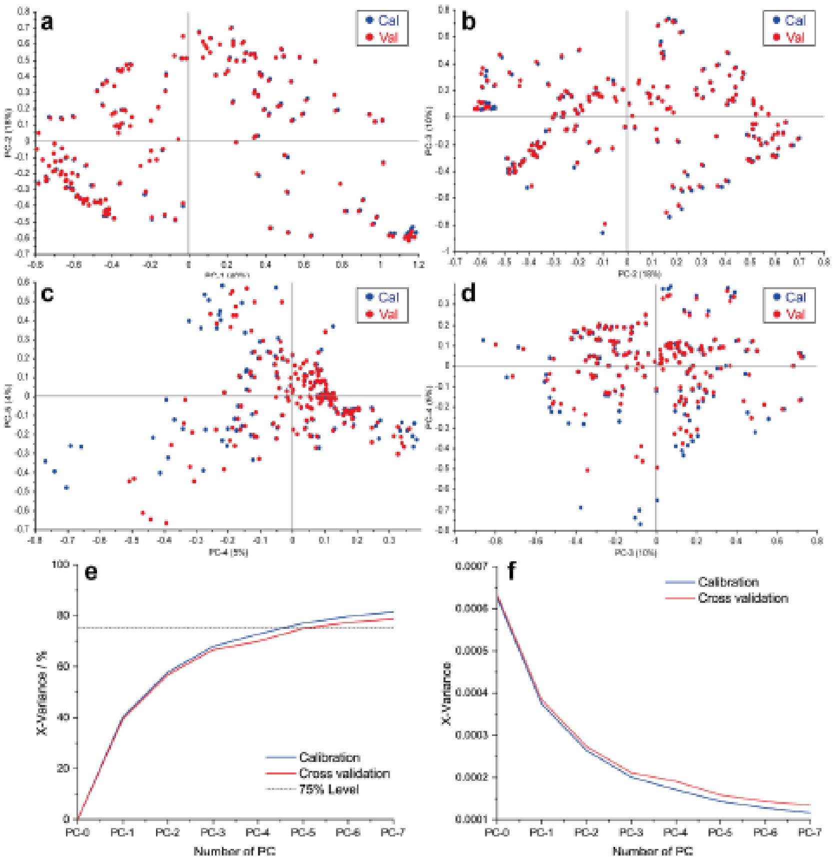

Figure S4. 2D score plots of the calibration and validation model and explained variance / residual variance of the PCA model (**a-f**). For a better readability, the 75% explained X-Variance was implied by the dotted line. Residual variance are shown as absolute values.

The DA was computed by using the PCA score values and the Mahalanobis distance algorithm with five PCs. As a result, the confusion matrix of the PCA-DA is obtained and presented in **Table S1**. Raman mean spectra assignments, summarized in the confusion matrix, represent an internal model validation, of which each model spectrum is allocated to one tissue cluster as if it was not actually included in the model. Based on this outcome, performance parameters can be calculated.

Table S1. Confusion matrix of the final PCA-DA model. An overall model accuracy of 94 % was achieved, which is also represented by the high number of correctly assigned model-included Raman mean spectra for all tissue types.

**Confusion matrix of the parotid tissue PCA-DA**		Actual		
		Salivary gland tissue	Pleomorphic adenoma	Warthin tumor
**Predicted**	Salivary gland tissue	91	3	3
	Pleomorphic adenoma	3	31	1
	Warthin tumor	0	1	43
